# The effect of pharmacist‐led interventions on the management and outcomes in chronic kidney disease (CKD): A systematic review and meta‐analysis protocol

**DOI:** 10.1002/hsr2.1064

**Published:** 2023-01-14

**Authors:** Ashkon Ardavani, Ffion Curtis, Kamlesh Khunti, Thomas J. Wilkinson

**Affiliations:** ^1^ NIHR Applied Research Collaboration East Midlands (ARC‐EM), Leicester Diabetes Center University of Leicester Leicester UK

**Keywords:** chronic kidney disease, interventions, meta‐analysis, pharmacist, randomized controlled trials, systematic review

## Abstract

**Background and Aims:**

Chronic kidney disease (CKD) is a progressive condition that results in a decline in kidney function over time. There are several conditions that increase the likelihood of developing CKD, particularly diabetes and hypertension. CKD increases the risk of mortality and has a detrimental impact on quality of life (QoL). Strategies for managing CKD include controlling cardiovascular risk factors and treating complications of CKD. There is an ever‐increasing role of pharmacists in managing CKD, from the optimization of risk factors to patient education. However, currently, there is a lack of data on the effect pharmacist‐led interventions have on the clinical, economic, and humanistic outcomes.

**Methods:**

This protocol, in adherence to PRISMA‐P (Preferred Reporting Items for Systematic review and Meta‐Analysis Protocols) standards, describes a prospective systematic review and meta‐analysis of randomized controlled trials, where any intervention led by a pharmacist in CKD is used. Comparison groups will consist of usual care or non‐pharmacist‐led interventions. Literature searches will be conducted in the following databases: MEDLINE, Scopus, and Web of Science. Data pertaining to clinical (e.g., mortality), economic (e.g., healthcare‐associated costs), and humanistic (e.g., QoL) outcomes will be extracted. Risk of bias will be assessed using the United States National Heart Lung and Blood Institute quality assessment tool for controlled intervention studies. A meta‐analysis will be conducted to synthesize appropriate comparable outcomes.

**Results:**

The findings of this review will be published in a peer‐reviewed journal, where the results will be presented in lay language with appropriate infographics online and via social media.

**Conclusion:**

The findings of this review can identify gaps in the literature concerning optimizing pharmacist‐led interventions in improving outcomes. In addition, this review will establish the importance of pharmacists in managing CKD patients, and whether this may result in their increased incorporation in multidisciplinary teams.

## 1 INTRODUCTION

Chronic kidney disease (CKD) is a condition characterized by the gradual change in the function and structure of kidneys over time.^1^ It is defined as a reduction in kidney function, with an estimated glomerular filtration rate (eGFR) of <60 ml/min per 1.73 m^2^ and/or markers of kidney damage for at least 3 months.^2^ Kidney replacement therapy (KRT), such as dialysis or kidney transplantation, is offered to patients whose kidney function has deteriorated and who have consequently developed kidney failure (i.e., an eGFR <15 ml/min per 1.73 m^2^).^3^ Whilst there are several aetiologies of CKD, including diabetes and hypertension,^4^ the prevalence of CKD increases with age.^5^ CKD has a prevalence of around 14% in those aged 30 years and over, increasing to 34% in people in over 70 years.^6^ Kidney function steadily declines over time, with a mean annual rate of change in eGFR of  approximately 1 ml/min per 1.73 m^2^.^7^ The loss of function can be attributed to reductions in afferent arteriolar resistance, glomerular capillary plasma flow rate and kidney mass, alongside other haemodynamic and structural changes.^8^ Multimorbidity is also becoming increasingly prevalent in older adults,^9^ where chronic conditions like diabetes and hypertension often coexist and can further increase the risk of CKD.^10^ CKD can have a significantly detrimental impact on quality of life (QoL) and can result in adverse health outcomes such as an increased risk of cardiovascular disease,^11^ anaemia, and mineral and bone disorders.^12^

Effective identification and management of the condition, which is primarily conducted in primary care,^13^ is essential. Management of CKD patients is multifactorial, where the aims are to identify and control risk factors such as hypertension with the use of renin‐angiotensin aldosterone system (RAAS) inhibitors, angiotensin‐converting enzyme inhibitors or angiotensin receptor antagonists, lipid reduction with the use of statins and various glucose‐lowering pharmacological agents for diabetes.^1^ Risk factor optimization is part of a holistic approach towards CKD management, alongside lifestyle modifications, the treatment of comorbidities, complications and symptoms, and reduction in the progression of eGFR decline with the use of pharmacological therapies such as RAAS inhibitors and sodium‐glucose cotransporter‐2inhibitors.^1^ Patients who reach an advanced stage of CKD require a multidisciplinary approach to manage their symptoms and improve their QoL, where dialysis or kidney transplantation can be offered as options for KRT.^1^, ^14^ Polypharmacy is prevalent in kidney transplant patients, where they often take immunosuppressants and medications to prevent graft rejection and to manage other comorbidities.^15^ Where KRT is not appropriate, conservative care can be offered, which consists of symptom management, the provision of palliative care, and advance‐care planning.^16^

Pharmacists provide a valuable contribution in managing CKD patients and improving their clinical, economic, and humanistic outcomes, either individually or as part of a wider multidisciplinary team. A 2‐year prospective cohort study by Leung et al.^17^ that compared structured care provided by a pharmacist‐diabetes specialist team to usual care resulted in a reduction in the incidence of end‐stage kidney disease or death in patients with type 2 diabetic nephropathy. Pharmacists can primarily help in managing drug‐related problems. A retrospective study by Daifi et al.^18^ demonstrated that a clinical pharmacist who conducted medication reconciliation and medication reviews in haemodialysis patients identified over 1400 drug‐related problems and saved approximately US$450,000. Pharmacists may also provide routine care of risk factor control and self‐management education programmes. Pharmacists have also played a crucial role in managing CKD patients during the COVID‐19 pandemic, from identifying and preventing potential or actual drug‐drug interactions to making dose adjustments or optimizing doses of medication indicated for COVID‐19, as per the patient's kidney function.^19^ There is growing evidence that pharmacists can improve outcomes in patients with CKD and there remains a growing opportunity to improve their incorporation into CKD healthcare teams.^20^ Nonetheless, data on how pharmacists are most effectively used in the management of CKD is limited, and optimizing interventions delivered by pharmacists may help improve the care of people living with CKD.

The purpose of this review is to identify pharmacist‐led interventions in management of CKD, including dialysis and nondialysis patients, and the effect on clinical, economic, and humanistic outcomes, compared to usual care or appropriate comparison group (e.g., nonpharmacist intervention). Whilst several previous reviews have been undertaken exploring the effect of pharmacist interventions in CKD, they have included various types of research study designs and had a narrow search strategy,^21^, ^22^, ^23^ where they had a limited number of search terms and did not have any for the key concept of “intervention.” Moreover, two of these systematic reviews are out‐of‐date,^22^, ^23^ where research has been conducted since their publication as recently as 2021, with two randomized controlled trials (RCTs) that investigated the impact of pharmacist‐led mobile health interventions in kidney transplant patients.^24^, ^25^ This contemporary review will have a broader search strategy encompassing a range of outcomes to provide a comprehensive overview of pharmacist interventions in CKD. Furthermore, it will include only RCTs, the highest level of evidence,^26^ and a meta‐analysis will aim to synthesize appropriate comparable outcomes.

## 2 METHODS

The systematic review protocol is registered on the International Prospective Register of Systematic Reviews (PROSPERO) (Registration number: CRD42022304902). The protocol has been developed in adherence to PRISMA‐P (Preferred Reporting Items for Systematic review and Meta‐Analysis Protocols) standards^27^ (see Supporting Information Material S1).

### 2.1 Eligibility criteria

Inclusion and exclusion criteria can be found in Table 1.

**Table 1:** Inclusion and exclusion criteria

Inclusion criteria	Exclusion criteria
Any form of RCT, including clustered controlled trials and any trial where participants are randomized to receive an intervention(s) against an appropriate comparator group Wait‐list or cross‐over study designs Adults that are aged 18 years or over with a diagnosis of CKD, including those with nondialysis CKD and those receiving (in recipient of) kidney replacement therapy, such as dialysis or transplantation CKD in the presence of other long term‐conditions Studies in English language	Abstracts/conference proceedings Protocols Seminars Case‐control studies Case reports Cohort studies Case series Cross‐sectional studies Adolescents and children below the age of 18 years and animals Non‐English studies

Abbreviations: CKD, chronic kidney disease; RCT, randomized controlled trial.

### 2.2 Information sources

Searches of existing and completed systematic reviews will be conducted on the Cochrane Library and PROSPERO databases, and we will screen their reference lists to identify any potential eligible studies. Literature searches will be conducted in the following databases: MEDLINE, Scopus, and Web of Science. A combination of free‐text terms and medical subject heading (MESH) terms will be used to search MEDLINE, whereas only free‐text searching will be used for Scopus and Web of Science. Database searches will be supplemented with internet searches (i.e., Google Scholar) and forward and backward citation tracking from included studies and review articles. There will be no restrictions on when the relevant studies identified were published. Key search terms that were used in Clarivate Analytics Web of Science include pharmacist* (Topic) or “Pharmacy Service, Hospital” (Topic) AND intervention* (Topic) or “Medic* management” (Topic) AND “Chronic kidney disease” (Topic) or CKD (Topic) (see Supporting Information Material 1 for an example search strategy). The search terms were developed with support from the library research services consultant at the University of Leicester.

### 2.3 Selection process

Results from the searches will be combined in EndNote 20 (Clarivate Analytics), where duplicates will be removed before uploading retrieved references to the Covidence systematic review management system (www.covidence.org).^28^ Two independent reviewers will screen initial title and abstracts for inclusion. Any uncertainties or disagreements that arise will be resolved with input from a third reviewer. Full texts will be obtained for references not excluded during title and abstract screening and will be independently screened by two reviewers.

### 2.4 Data collection process

One reviewer will extract data and a second will check for accuracy. A bespoke extraction form will be created to document extracted information from the studies. The form will be piloted and amended, if necessary, before full extraction.

### 2.5 Data items

The extracted information will include the following: sociodemographic (e.g., age, sex and ethnicity) and clinical characteristics (eGFR, systolic blood pressure and diastolic blood pressure); a description of details of the intervention and control groups such as sample size, type of intervention and duration; primary and secondary outcomes; and key conclusions from the authors. In addition, information will be extracted on the resources available for pharmacists for provision of care and the processes they carry out.

### 2.6 Outcomes and prioritization

Data will be extracted based on economic (e.g., cost of inappropriately prescribed medications and hospitalization‐associated costs), humanistic outcomes (e.g., QoL and patient knowledge) and clinical (e.g., blood pressure and mortality) measures (see Table 2 for definition of clinical, economic, and humanistic outcomes).

**Table 2:** Outcomes of interest

Outcome category	Definition	Examples
Clinical	Medical events that occur due to disease or treatment^29^	SBP and eGFR
Economic	The direct, indirect, and intangible costs in comparison to the consequences of alternative medical treatments^29^	Healthcare‐associated costs and costs of inappropriately prescribed medications
Humanistic	The outcomes of disease or treatment on the functional status or QoL of a patient^29^	Patient knowledge and well‐being

Abbreviations: eGFR, estimated glomerular filtration rate; QoL, quality of life; SBP, systolic blood pressure.

### 2.7 Risk of bias in individual studies

Two reviewers will independently assess the risk of bias using the United States National Heart Lung and Blood Institute (US NHLBI) quality assessment tool for controlled intervention studies.^30^ If there is a disagreement between them, input from a third reviewer will be provided to reach a consensus. In addition, an Egger's test will be used to identify potential publication bias.^31^

### 2.8 Data synthesis

A narrative synthesis will be created from the included studies structured around the type of intervention, target population characteristics and outcome measures. Where appropriate (e.g., comparable outcomes across studies), Review Manager (RevMan Version 5.4.1) software (The Cochrane Collaboration) will be used to analyse data, where continuous data will be expressed as a mean or standardized mean difference with a 95% confidence interval and dichotomous outcomes will be presented as either relative risk or odds ratio. Data will not be pooled if heterogeneity is moderate (*I* ^2^ statistic greater than 40%).^32^ Moreover, the level of significance will be set at <0.05. If heterogeneity is identified, potential causes will be explored (e.g., clinical and/or methodological diversity). We will explore heterogeneity via subgroup analysis, but if this cannot be explained, then a narrative approach will be taken, and a meta‐analysis will not be performed. Studies with a moderate or high risk of bias will be removed from analyses to determine the extent to which synthesized results are sensitive to risk of bias.

### 2.9 Analysis of subgroups or subsets

If possible, we will conduct subgroup analyses across the different disease staging, (e.g., nondialysis, dialysis, transplantation), the type of pharmacist‐led interventions, the presence of comorbidities, if interventions delivered were by a multidisciplinary team including a pharmacist or were pharmacist‐led, and by sociodemographic groupings (e.g., sex, ethnicity, age).

## 3 DISCUSSION

CKD is an age‐related disease characterized by the loss of kidney function or markers of kidney damage for at least 3 months. CKD prevalence increases with age, and often exists in the presence of other comorbidities such as hypertension and diabetes. The management of CKD involves the identification and treatment of risk factors, comorbidities, symptoms, complications, as well as reducing the decline in eGFR. Pharmacists are well placed with their clinical and therapeutic knowledge to provide pharmaceutical and nonpharmaceutical recommendations and interventions for CKD patients. The aim of this systematic review and meta‐analysis is to explore the impact of pharmacist‐led interventions in managing clinical, economic, and humanistic outcomes of CKD patients enrolled in RCTs. The findings of this review will be published in a peer‐reviewed journal, where the results will be presented in lay language with appropriate infographics online and via social media. Moreover, any potential gaps in the literature may be identified with regards to pharmacist interventions, where information from this review will be utilized to ascertain what aspects of interventions delivered by a pharmacist (e.g., patient education) were able to improve clinical, economic and/or humanistic outcomes. Consequently, trials can be devised to optimize and address them, alongside identifying alternative models of care that may help with redesigning services to manage people with CKD. In addition, this review will look to establish the importance of pharmacists in managing CKD patients compared to those receiving usual care, and whether this potentially will result in increased incorporation of pharmacists in multidisciplinary teams and/or more interventions delivered solely by the pharmacist which will optimize practice in the future.

## AUTHOR CONTRIBUTIONS

**Ashkon Ardavani**: conceptualization; methodology; writing—original draft; writing—review & editing. **Ffion Curtis**: conceptualization; methodology; supervision; writing—review & editing. **Kamlesh Khunti**: conceptualization; methodology; supervision; writing—review & editing. **Thomas J Wilkinson**: conceptualization; methodology; supervision; writing—review & editing. All authors provided critical revisions for important intellectual content. All authors have read and approved the final version of the manuscript. All authors agreed to be accountable for all aspects of the work.

## CONFLICTS OF INTEREST

The authors declare no conflicts of interest.

## ETHICS STATEMENT

An ethics statement is not applicable because this study is based exclusively on published literature.

## TRANSPARENCY STATEMENT

The lead author Ashkon Ardavani affirms that this manuscript is an honest, accurate, and transparent account of the study being reported; that no important aspects of the study have been omitted; and that any discrepancies from the study as planned (and, if relevant, registered) have been explained.

## Supporting information

Supporting information.

## Data Availability

Data will be made available upon reasonable request. Further enquiries can be directed to the corresponding author. TJW have full access to all of the data in this study and takes complete responsibility for the integrity of the data and the accuracy of the data analysis.

## References

[1] Kalantar‐Zadeh K , Jafar TH , Nitsch D , Neuen BL , Perkovic V . Chronic kidney disease. The Lancet. 2021;398(10302):786‐802. 10.1016/S0140-6736(21)00519-5 34175022

[2] Kidney Disease: Improving Global, Outcomes (KDIGO) CKD Work Group . KDIGO 2012 clinical practice guideline for the evaluation and management of chronic kidney disease. Kidney Int Suppl. 2013;3(1):1‐150.

[3] NICE | The National Institute for Health and Care Excellence. Recommendations | Chronic kidney disease: assessment and management | Guidance | NICE [Internet]. 2021 [cited 2022 Mar 21]. Available from: https://www.nice.org.uk/guidance/ng203/chapter/Recommendations

[4] Webster AC , Nagler EV , Morton RL , Masson P . Chronic kidney disease. The Lancet. 2017;389(10075):1238‐1252. 10.1016/S0140-6736(16)32064-5 27887750

[5] Tonelli M , Riella M . Chronic kidney disease and the aging population. Am J Nephrol. 2014;39(3):248‐251. 24642456 10.1159/000359957

[6] Hill NR , Fatoba ST , Oke JL , et al. Global prevalence of chronic kidney disease—a systematic review and meta‐analysis. PLoS One. 2016;11(7):e0158765. 27383068 10.1371/journal.pone.0158765 PMC4934905

[7] Turin TC , Coresh J , Tonelli M , et al. Change in the estimated glomerular filtration rate over time and risk of all‐cause mortality. Kidney Int. 2013;83(4):684‐691. 23344477 10.1038/ki.2012.443

[8] Weinstein JR , Anderson S . The aging kidney: physiological changes. Adv Chronic Kidney Dis. 2010;17(4):302‐307. 20610357 10.1053/j.ackd.2010.05.002 PMC2901622

[9] Salive ME . Multimorbidity in older adults. Epidemiol Rev. 2013;35(1):75‐83. 23372025 10.1093/epirev/mxs009

[10] Jindal A , Whaley‐Connell A , Sowers JR . Type 2 diabetes in older people; the importance of blood pressure control. Current Cardiovascular Risk Reports. 2013;7(3):233‐237. 23667714 10.1007/s12170-013-0301-5 PMC3647695

[11] Jankowski J , Floege J , Fliser D , Böhm M , Marx N . Cardiovascular disease in chronic kidney disease. Circulation. 2021;143(11):1157‐1172. 33720773 10.1161/CIRCULATIONAHA.120.050686 PMC7969169

[12] Bello AK , Alrukhaimi M , Ashuntantang GE , et al. Complications of chronic kidney disease: current state, knowledge gaps, and strategy for action. Kidney Int Suppl. 2017;7(2):122‐129. 10.1016/j.kisu.2017.07.007 PMC6341007 30675426

[13] Bello AK , Johnson DW . Educating primary healthcare providers about kidney disease. Nature Reviews Nephrol. 2021;18:133‐134. 10.1038/s41581-021-00527-y PMC8679563 34921298

[14] Sakhuja A , Hyland J , Simon JF . Managing advanced chronic kidney disease: a primary care guide. Cleve Clin J Med. 2014;81(5):289‐299. 24789588 10.3949/ccjm.81a.13046

[15] Marienne J , Laville SM , Caillard P , et al. Evaluation of changes over time in the drug burden and medication regimen complexity in ESRD patients before and after renal transplantation. Kidney Int Rep. 2021;6(1):128‐137. 33426392 10.1016/j.ekir.2020.10.011 PMC7785410

[16] Vaidya SR , Aeddula NR Chronic Renal Failure. StatPearls Publishing; 2021.

[17] Leung WYS , So WY , Tong PCY , Chan NN , Chan JCN . Effects of structured care by a pharmacist‐diabetes specialist team in patients with type 2 diabetic nephropathy. Am J Med. 2005;118(12):1414.e21‐1414.e27. 10.1016/j.amjmed.2005.07.050 16378791

[18] Daifi C , Feldpausch B , Roa PA , Yee J . Implementation of a clinical pharmacist in a hemodialysis facility: a quality improvement report. Kidney Medicine. 2021;3(2):241‐247. 33851119 10.1016/j.xkme.2020.11.015 PMC8039404

[19] Karattuthodi MS , Thorakkattil SA , Abdulsalim S , et al. The pharmacist's role in managing COVID‐19 in chronic kidney disease patients: a review of existing strategies and future implications. Pharmacy (Basel, Switzerland). 2022;10(4):94. 36005934 10.3390/pharmacy10040094 PMC9412434

[20] St Peter WL , Wazny LD , Patel UD . New models of CKD care including pharmacists: improving medication reconciliation and medication management. Curr Opin Nephrol Hypertens. 2013;22(6):662. 10.1097/MNH.0b013e328365b364 PMC4012859 24076556

[21] Al Raiisi F , Stewart D , Fernandez‐Llimos F , Salgado TM , Mohamed MF , Cunningham S . Clinical pharmacy practice in the care of chronic kidney disease patients: a systematic review. Int J Clin Pharm. 2019;41(3):630‐666. 30963447 10.1007/s11096-019-00816-4 PMC6554252

[22] Stemer G , Lemmens‐Gruber R . Clinical pharmacy activities in chronic kidney disease and end‐stage renal disease patients: a systematic literature review. BMC Nephrol. 2011;12(35):35. 21777480 10.1186/1471-2369-12-35 PMC3166893

[23] Salgado TM , Moles R , Benrimoj SI , Fernandez‐Llimos F . Pharmacists' interventions in the management of patients with chronic kidney disease: a systematic review. Nephrol Dial Transplant. 2012;27(1):276‐292. 21719712 10.1093/ndt/gfr287

[24] Gonzales HM , Fleming JN , Gebregziabher M , et al. Pharmacist‐led mobile health intervention and transplant medication safety: a randomized controlled clinical trial. Clin J Am Soc Nephrol. 2021;16(5):776‐784. 33931415 10.2215/CJN.15911020 PMC8259471

[25] Fleming JN , Gebregziabher M , Posadas A , Su Z , Mcgillicuddy JW , Taber DJ . Impact of a pharmacist‐led, mHealth‐based intervention on tacrolimus trough variability in kidney transplant recipients: a report from the TRANSAFE Rx randomized controlled trial. Am J Health Syst Pharm. 2021;78(14):1287‐1293. 33821958 10.1093/ajhp/zxab157 PMC8599187

[26] Burns PB , Rohrich RJ , Chung KC . The levels of evidence and their role in evidence‐based medicine. Plast Reconstr Surg. 2011;128(1):305‐310. 21701348 10.1097/PRS.0b013e318219c171 PMC3124652

[27] Shamseer L , Moher D , Clarke M , et al. PRISMA‐P (Preferred Reporting Items for Systematic review and Meta‐Analysis Protocols) 2015 checklist: recommended items to address in a systematic review protocol* [Internet]. 2015 [cited 2022 Mar 4]. Available from: http://www.prisma-statement.org/documents/PRISMA-P-checklist.pdf

[28] Covidence. Covidence ‐ Better systematic review management [Internet]. [cited 2022 Mar 22]. Available from: https://www.covidence.org/

[29] Barry HE , Hughes CM . Economical, clinical, and humanistic outcomes and pharmaceutical care. In: da Costa FA , van Mil JWF , Alvarez‐Risco A , editors, The Pharmacist Guide to Implementing Pharmaceutical Care. Springer; 2019:119‐127.

[30] NHLBI N Study Quality Assessment Tools ‐ Quality Assessment of Controlled Intervention Studies [Internet]. 2021 [cited 2022 Nov 29]. Available from: https://www.nhlbi.nih.gov/health-topics/study-quality-assessment-tools

[31] Peters JL . Comparison of two methods to detect publication bias in meta‐analysis. JAMA. 2006;295(6):676‐680. 16467236 10.1001/jama.295.6.676

[32] Deeks J , Higgins J , Altman DG Chapter 10: analysing data and undertaking meta‐analyses [Internet]. 2022 [cited 2022 Mar 21]. Available from: https://training.cochrane.org/handbook/current/chapter-10#section-10-10

